# The Relationship between Sarcopenia and Systemic Inflammatory Response for Cancer Cachexia in Small Cell Lung Cancer

**DOI:** 10.1371/journal.pone.0161125

**Published:** 2016-08-18

**Authors:** Eun Young Kim, Young Saing Kim, Ja-Young Seo, Inkeun Park, Hee Kyung Ahn, Yu Mi Jeong, Jeong Ho Kim, Nambeom Kim

**Affiliations:** 1 Department of Radiology, Gachon University Gil Medical Center, Incheon, Republic of Korea; 2 Division of Hematology and Oncology, Department of Internal Medicine, Gachon University Gil Medical Center, Incheon, Republic of Korea; 3 Department of Laboratory Medicine, Gachon University Gil Medical Center, Incheon, Republic of Korea; 4 Neuroscience Research Institute, Gachon University, Incheon, South Korea; University of Nebraska Medical Center, UNITED STATES

## Abstract

**Background:**

The prognostic significance of sarcopenia, an important component of cancer cachexia, has been demonstrated in oncologic patients. Catabolic drivers have been suggested to be key features of cancer cachexia.

**Objective:**

To determine the relationship between systemic inflammatory markers and CT-determined muscle mass in patients with SCLC.

**Methods:**

Cross-sectional muscle areas were evaluated at the level of the third lumbar vertebra (L3) using baseline CT images in 186 SCLC patients. Sarcopenia was defined as a L3 muscle index (L3MI, muscle area at L3/height^2^) of < 55 cm^2^/m^2^ for men and of < 39 cm^2^/m^2^ for women. Systemic inflammatory markers investigated included serum white blood cell count (WBC), neutrophil: lymphocyte ratio (NLR), C-reactive protein (CRP), and albumin.

**Results:**

Mean L3MI was 47.9 ± 9.7 cm^2^/m^2^ for men and 41.6 ± 7.0 cm^2^/m^2^ for women. Sarcopenia was present in 128 patients (68.8%), and sarcopenic patients had significant serum lymphocyte counts and albumin levels (*p* = 0.002 and 0.041, respectively), and higher NLRs and CRP levels (*p* = 0.011 and 0.026) than non-sarcopenic patients. Multivariable analysis revealed that CRP independently predicted L3MI (*β* = -0.208; 95% CI, -0.415 to -0.002; *p* = 0.048), along with gender and BMI (*p* values < 0.001) and performance status (*p* = 0.010).

**Conclusion:**

The present study confirms a significant linear relationship exists between CT-determined muscle mass and CRP in SCLC patients. This association might provide a better understanding of the mechanism of cancer cachexia.

## Introduction

Lung cancer is the leading cause of cancer death worldwide and small cell lung cancer (SCLC) accounts for about 15% of all cases. Although SCLC is highly responsive to initial chemotherapy and radiotherapy, its prognosis is poor [1, 2]. A number of clinical indicators are related to prognosis in SCLC. Tumor staging systems are the most important predictors of overall survival (OS), and some studies have indicated clinical characteristics, such as, gender, age, smoking status, and performance status also have prognostic significance [1, 3]. Depletion of muscle mass (sarcopenia) is prevalent in lung cancer patients, and recently, a study revealed its prognostic significance in SCLC [4]. In fact, sarcopenia is considered an important component of cancer cachexia syndrome, and its prognostic significance in the oncologic setting emphasizes the need for better understanding of its mechanisms and the need for effective therapeutic interventions to increase muscle mass.

Hyper-catabolism caused by tumor metabolism, systemic inflammation, and other tumor-mediated effects have been suggested to be the key features of cancer cachexia syndrome. Population-based studies suggest inflammatory response is involved in age-related muscle mass reduction [5, 6]. CT scan is objective and precise measure to quantify skeletal muscle mass, and a direct relationship was found between low skeletal muscle mass determined by CT and the presence of a systemic inflammatory response in operable colorectal cancer patients [7]. However, little is known about the relationship between sarcopenia determined by CT and systemic inflammatory response in SCLC patients. Accordingly, the primary objective of this study was to evaluate the relationship between systemic inflammatory markers and skeletal muscle mass, as determined by baseline CT images, in SCLC patients.

## Methods

### Patients

The radiology database and medical records at Gachon University Gil Medical Center (Incheon, Korea) were searched for patients with newly diagnosed, pathologically proven SCLC that underwent a baseline chest CT and PET/CT scan from January 2010 to October 2015. Heights and weights were measured and functional statuses recorded at first visit to our oncology department. Body mass index (BMI) was defined as weight divided by height squared (kg/m^2^), and BMI was categorized as underweight (< 18.5 kg/m^2^), normal (18.5–22.9 kg/m^2^), overweight (23.0–24.9 kg/m^2^), or obese (≥ 25.0 kg/m^2^) [8].

SCLC was classified as limited or extensive. Limited stage was defined as American Joint Committee on Cancer (AJCC) stages I to III, which can be safely treated by definitive radiation therapy [9]. The institutional review board of our hospital approved this retrospective study and waived the requirement for informed patient consent.

### Serum inflammatory markers

Pretreatment venous blood samples were collected after a 12-hour overnight fast and used to determine WBC, neutrophil and lymphocyte counts, and serum albumin, lactate dehydrogenase (LDH) and C-reactive protein (CRP) levels. Blood cell counts were measured using an ADVIA 2120 Hematology System (Siemens AG, Eschborn, Germany) using whole blood collected in EDTA tube (Becton Dickinson, Franklin lakes, NJ, USA). Serum specimens were generated from whole blood collected in serum separating tube (Becton Dickinson, USA) by centrifuging at 1,500g for 10 minutes. Albumin, LDH, and CRP were determined from fresh serum specimens using an ADVIA 2400 Chemistry System (Siemens Healthcare Diagnostics, Sacramento, CA, USA).

Systemic inflammatory markers included neutrophil to lymphocyte ratio (NLR) and modified Glasgow Prognostic Score (mGPS; a combination of CRP and albumin). The mGPS was scored as follows [10]; patients with a normal CRP (≤ 10 mg/L) and albumin (≥ 35 g/L) were allocated a score of 0, patients with high CRP and low albumin were scored as 2, and patients with high CRP only were scored as 1.

### Image analysis

Single cross-sectional areas of third lumbar vertebrae (L3MA), determined using PET/CT images obtained at time of SCLC diagnosis, served as reference standard. A six-detector CT (Siemens Medical Systems, Erlangen, Germany), equipped with lutetium oxyorthosilicate crystal PET detectors, was used with the following imaging parameters; 130 kVp, 110 mAs, 2-mm pitch, 1-s tube rotation, and slice thickness 5 mm, which matched the section thickness of PET images.

**Fig 1:**
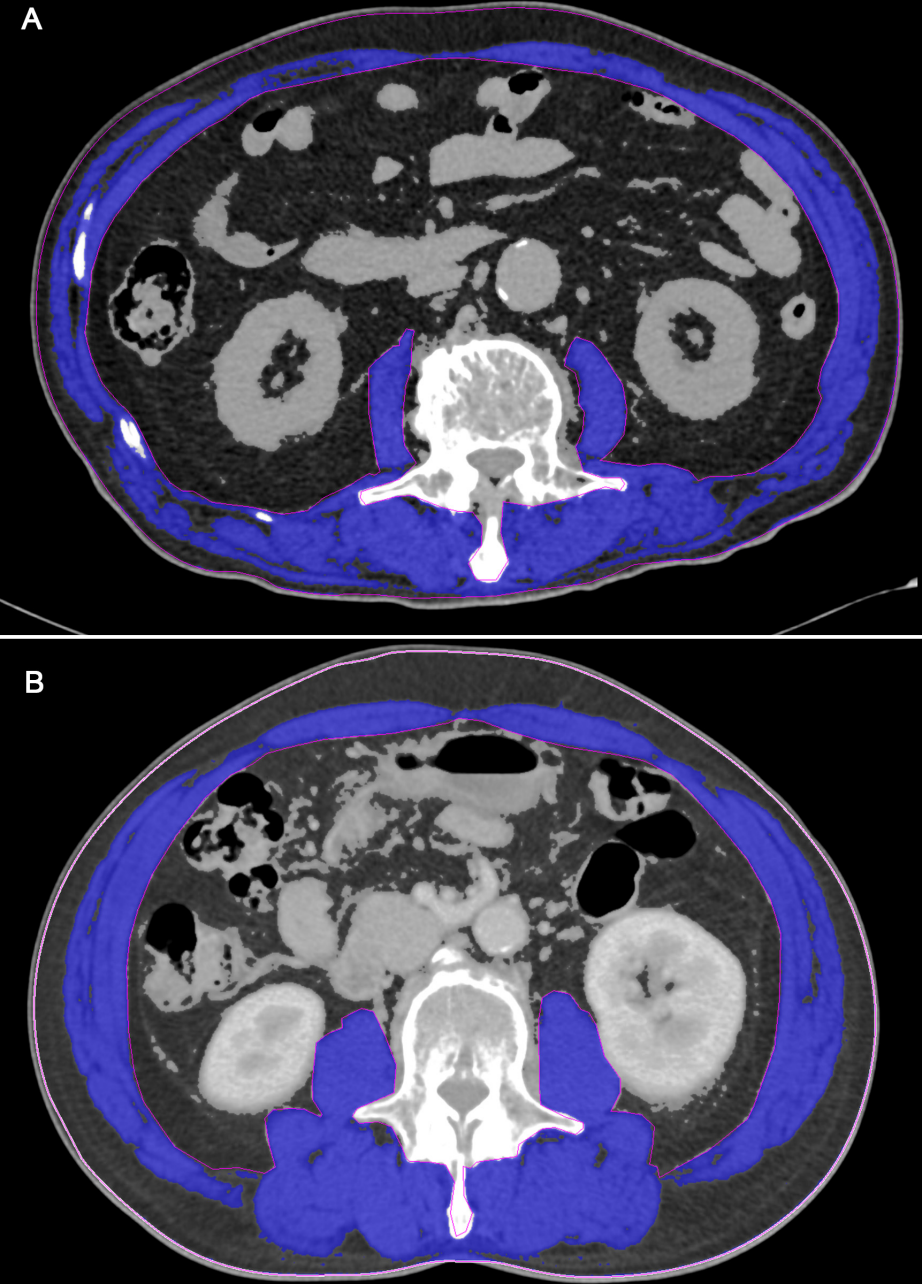
Single cross-sectional area of skeletal muscle at the third lumbar vertebrae and the level of C-reactive protein (CRP) in patients with small cell lung cancer. (a) In a 72-year-old male patients, the CT-measured L3 muscle (blue color) index was 43.4 cm^2^/m^2^ and the CRP was 11.58 mg/dL at the time of diagnosis. (b) In a 69-year-old male patients, the CT-measured L3 muscle (blue color) index was 75.0 cm^2^/m^2^ and the CRP was 0.09 mg/dL at the time of diagnosis.

Quantitative assessments of muscle areas were performed using commercially available software (Terarecon 3.4.2.11, San Mateo, CA, USA) by a subspecialty-trained chest radiologist. Tissue cross-sectional areas (cm^2^) of respective tissues in slices were computed automatically by summing appropriate pixels using the CT Hounsfield unit (HU) ranges -29 HU to 150 HU for skeletal muscle. After applying threshold methods using a predefined HU threshold set for each slice, boundaries between different tissues were corrected manually when necessary (Fig 1). L3 muscle index (L3MI, cm^2^/m^2^) was defined as the cross-sectional area of muscle at the L3 level normalized for stature as is conventional for BMI.

Sarcopenia was defined as a L3MI of < 55 cm^2^/m^2^ for men and of < 39 cm^2^/m^2^ for women, as proposed by international consensus for cancer cachexia [1].

### Statistical analysis

Descriptive statistics are reported as proportions or means with standard deviations (SDs). For categorical variables, comparisons between subjects with and without sarcopenia were performed using Pearson’s chi-squared test or Fisher’s exact test. Continuous variables were compared using the independent two-sample t-test for the variables with normal distributions; otherwise, nonparametric Mann-Whitney U test was used.

The association between systemic inflammatory markers and height-adjusted muscle mass (L3MI) were determined by partial correlation analysis controlling for gender, age, weight, and BMI. In order to find the importance predictors for multivariable model, stepwise method with *F*—statistic were applied where candidate variables with a *p* value < 0.05 were entered, and those with a *p* value > 0.10 were removed from the model. In all assessment, *p* value ≤ 0.05 was considered statistically significant. The analysis was performed using SPSS for Windows ver. 19.0 (SPSS Inc., Chicago, IL, USA).

## Results

### Characteristics of the study population

A total of 186 consecutive patients were included in the present study (Table 1). Mean patient age was 68.6 ± 9.4 years and 156 (83.9%) were male. Of the 186 patients, 122 (65.6%) had extensive disease at first presentation. Average BMI was 22.3 ± 3.6 kg/m^2^ and 15.1% of the patients were underweight (n = 28). Mean L3MI was 49.6 ± 9.6 cm^2^/m^2^ for men and 43.7 ± 7.9 cm^2^/m^2^ for women.

**Table 1:** Characteristics of patients with small cell lung cancer according to the presence of sarcopenia.

Characteristics	All (n = 186)	Sarcopenia (n = 128)	No sarcopenia (n = 58)	*p* value
**Age, years**	68.8 ± 9.4	69.4 ± 9.5	67.6 ± 9.0	0.237^a^
**≥ 65 years**	127 (67.1%)	89 (69.5%)	38 (65.5%)	0.586^b^
**Male**	156 (83.9%)	119 (93.0%)	37 (63.8%)	< 0.001^b^
**Smoking Status**				
**Current/ex-smoker**	166 (89.2%)	116 (90.6%)	50 (86.2%)	0.368^b^
**Never smoker**	20 (10.8%)	12 (9.4%)	8 (13.8%)	
**Pack year, median (range)**	35 (0–171)	40 (0–171)	34 (0–120)	0.309^c^
**Stage**				
**Limited disease**	64 (34.4%)	39 (30.5%)	25 (43.1%)	0.093^b^
**Extensive disease**	122 (65.6%)	89 (69.5%)	33 (56.9%)	
**ECOG PS**				
**0–1**	132 (71.0%)	87 (68.0%)	45 (77.6%)	0.049^b^
**≥ 2**	54 (29.0%)	41 (32.0%)	13 (22.4%)	
**Charlson comorbidity index**				
**0**	59 (31.7%)	41 (32.0%)	18 (31.0%)	0.987^b^
**1–2**	101 (54.3%)	69 (53.9%)	32 (55.2%)	
**≥ 3**	26 (14.0%)	18 (14.1%)	8 (13.8%)	
**Body mass index, kg/m**^**2**^	22.3 ± 3.6	21.4 ± 3.3	24.3. ± 3.5	< 0.001^a^
**Underweight**	28 (15.1%)	26 (20.3%)	2 (3.4%)	< 0.001^b^
**Normal weight**	79 (42.5%)	61 (47.7%)	18 (31.0%)	
**Overweight**	40 (21.5%)	22 (17.2%)	18 (31.0%)	
**Obesity**	39 (21.0%)	19 (14.8%)	20 (34.5%)	
**Inflammatory markers**				
**WBC, 10**^**9**^**/L**	7.90 (3.58–24.11)	7.77 (3.58–24.11)	8.07 (3.92–15.02)	0.473^c^
**Neutrophil count, 10**^**9**^**/L**	5.00 (1.85–20.08)	5.04 (1.85–20.08)	4.92 (2.07–10.81)	0.646^c^
**Lymphocyte count, 10**^**9**^**/L**	1.74 (0.31–4.41)	1.69 (0.31–3.85)	2.04 (0.90–4.41)	0.002^c^
**Neutrophil to lymphocyte ratio**	2.7 (0.8–20.6)	3.07 (1.1–20.6)	2.50 (0.8–11.5)	0.011^c^
**CRP, mg/dL**	1.68 (0.01–29.03)	1.98 (0.02–29.02)	0.86 (0.01–22.85)	0.026^c^
**Albumin, g/dL**	3.95 (2.50–4.90)	3.90 (2.50–4.90)	4.10 (3.30–4.70)	0.041^c^
**mGPS score**				
**0**	160 (86.0%)	109 (85.2%)	51 (87.9%)	0.875^b^
**1**	19 (10.2%)	14 (10.9%)	5 (8.6%)	
**2**	7 (3.8%)	5 (3.9%)	2 (3.4%)	
**LDH (U/L), median (range)**	531 (282–8587)	544 (282–8587)	490 (294–2320)	0.111^c^
**Elevated LDH (≥ 486 U/L)**	105 (56.5%)	76 (59.4%)	29 (50.0%)	0.232^b^

Values are means ± standard deviations.

^a^ Student *t*- test

^b^ Chi-squared test

^c^Mann-Whitney *U* test

Abbreviations: ECOG PS, Eastern Cooperative Oncology Group performance status; CRP, C-reactive protein; mGPS, modified Glasgow Prognostic Score; LDH, lactate dehydrogenase

### Prevalence of factors associated with sarcopenia

The overall prevalence of sarcopenia was 68.8% (76.3% for men and 30.0% for women). In elderly patients (≥ 65 years old), the overall prevalence of sarcopenia was 70.1% (77.7% for men and 37.5% for women). The clinical characteristics of patients with or without sarcopenia are summarized in Table 1. Sarcopenia was found to be significantly associated with a male gender (*p* < 0.001), poor performance status (*p* = 0.049), and lower BMI (*p* < 0.001), and with inflammatory markers, that is, a high NLR (*p* = 0.011) and CRP (*p* = 0.026) and a low lymphocyte count (*p* = 0.002) and serum albumin level (*p* = 0.041). No significant difference was found between patients with or without sarcopenia in terms of age, smoking history, tumor stage, comorbidity index, elevated serum LDH (LDH ≥ 486 U/L), or mGPS.

### Relationships between L3MI and subject characteristics and systemic inflammatory markers

Correlations between L3MI and variables are summarized in Table 2. Weight, BMI, and smoking (pack years) were positively correlated with L3MI (r = 0.490, 0.534, and 0.152, respectively) but age and performance status were negatively associated with L3MI (r = -0.252 and -0.303, respectively). Lymphocyte count and albumin were positively correlated with L3MI (r = 0.151 and 0.243), whereas NLR and CRP showed negative correlations (r = -0.145 and -0.160).

**Table 2:** Correlations between L3 muscle index and patient’s characteristics and inflammatory markers.

Variables	*Correlation coefficients*	*p* value
**Age, year**	–0.252	0.001
**Gender, female**	-0.227	0.002
**ECOG PS**	-0.303	<0.001
**Weight, kg**	0.490	<0.001
**Body mass index, kg/m**^**2**^	0.534	<0.001
**Smoking, pack year**	0.152	0.039
**Charlson comorbidity index**	0.125	0.09
**Inflammatory markers**		
**Lymphocyte count**	0.151	0.039
**Neutrophil to lymphocyte ratio**	-0.145	0.048
**Albumin**	0.243	0.001
**CRP**	-0.16	0.029
**mGPS**	-.0.123	0.094

Abbreviations: ECOG PS, Eastern Cooperative Oncology Group performance status; CRP, C-reactive protein; mGPS, modified Glasgow Prognostic Score

Of the inflammatory markers, CRP and mGPS exhibited significant correlations with L3MI after adjusting for age, gender, BMI, and weight (partial correlation coefficients, -0.161 between CRP and L3MI, and -0.146 between mGPS and L3MI, *p* = 0.029 and 0.049, respectively).

**Fig 2:**
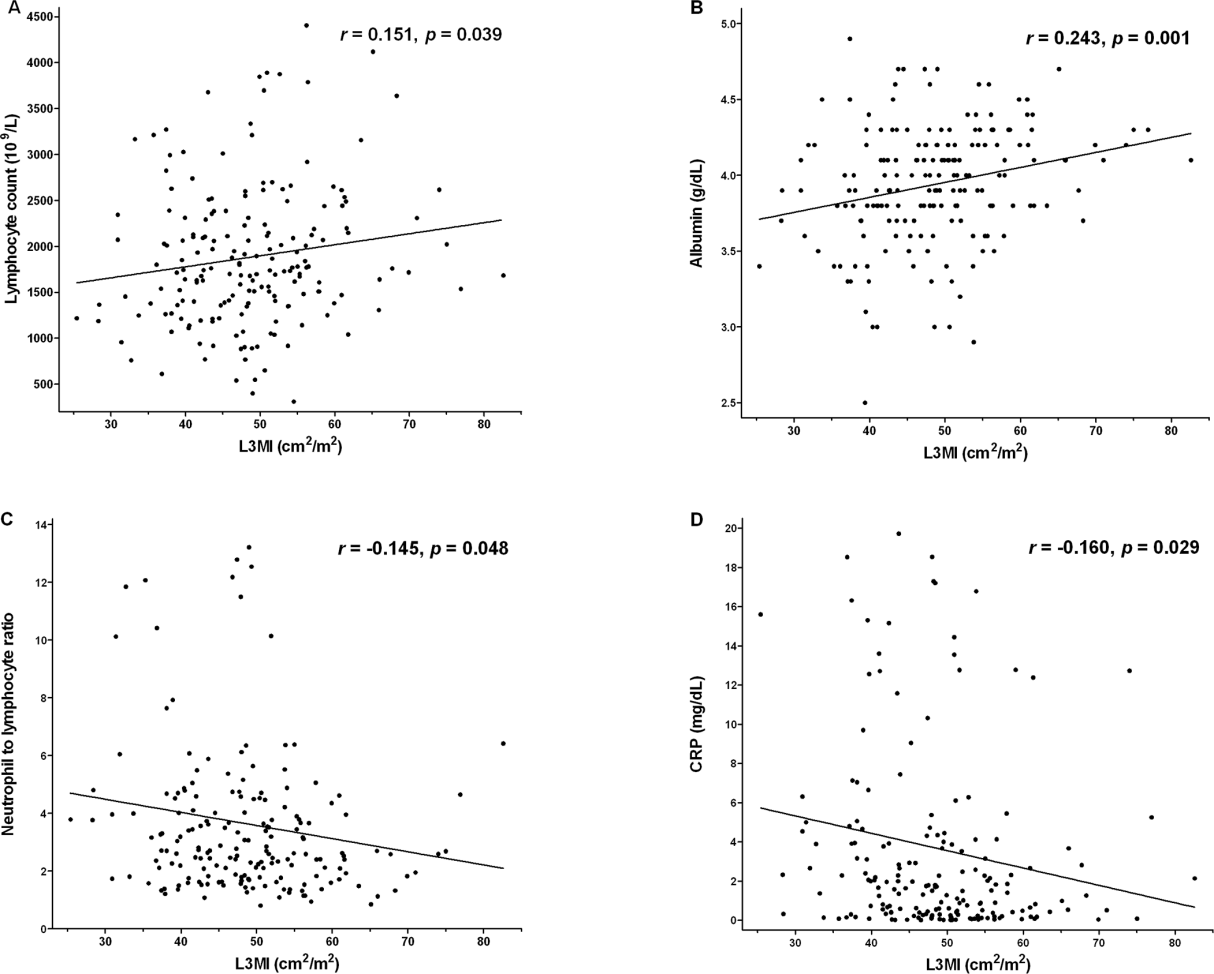
Correlation graphs between CT-determined L3 muscle index (L3MI) and inflammatory markers.

In the multivariable regression model, CRP was found to be the only significant inflammatory marker that predicted L3MI (*β* ***=*** -0.208; 95% confidence interval [CI], -0.415 to -0.002; *p* = 0.048) (Fig 2). Other variables that predicted L3MI were; female gender (*β* = -6.853; 95% CI, -9.835 to -3.872; *p* < 0.001), BMI (*β* = 1.344; 95% CI, 1.027 to 1.662; *p* < 0.001) and Eastern Cooperative Oncology Group (ECOG) performance status (*β* = -3.298; 95% CI, -5.796 to -0.799; *p* = 0.010) (Table 3).

**Table 3:** Multiple regression model to predict L3 muscle index (r = 0.851).

Variables	*β* ± SE	*p* value
**Gender, female**	-6.853 ± 1.511	< 0.001
**BMI, kg/m**^**2**^	1.344 ± 0.161	< 0.001
**ECOG PS ≥ 2**	-3.298 ± 1.266	0.010
**CRP**	-0.208 ± 0.105	0.048

Abbreviations: SE, standard errors; BMI, body mass index; ECOG PS, Eastern Cooperative Oncology Group performance status; CRP, C-reactive protein

## Discussion

Inflammation plays a key role in immune response by aiding the elimination of pathogens and the repair of tissue damage. However, inflammation can become chronic and promote the generations of reactive oxygen and nitrogen species and stimulate angiogenesis and cellular proliferation; all of which play key roles in carcinogenesis [11, 12]. Furthermore, accumulating epidemiological evidence implicates the involvement of systemic inflammation in cancer etiology and its prognostic significance in various cancers. mGPS (a compound variable of CRP and hypoalbuminemia) and NLR score (a systemic inflammation-based scoring system) have been shown to be of prognostic significance in different cancers including SCLC [3, 13–16].

Systemic inflammation also plays a significant role in the skeletal muscle depletion associated with cancer cachexia, which is a multifactorial metabolic syndrome characterized by ongoing loss of skeletal muscle mass with or without fat tissue loss [17–19]. Because decreased skeletal muscle mass is an independent predictor of immobility and mortality in advanced cancer, sarcopenia has become an important diagnostic component of this syndrome and an important therapeutic target [17, 20, 21]. The association between systemic inflammatory response and sarcopenia is supported by findings from experimental models, in which pro-inflammatory cytokines, including interleukin-1 (IL-1), IL-6, and tumor necrosis factor-α (TNF) were found to be mediators of anorexia and skeletal muscle proteolysis, the key components of cancer cachexia [22].

Serum CRP is the most widely accepted index of systemic inflammation because of its high sensitivity, specificity and reproducibility in hospital laboratories. It is interesting that albumin decreases as CRP increases in different tumor types [13–15], which explains the prognostic value of mGPS in various cancers. Albumin concentrations generally reflect nutritional status and amount of fat-free tissue [23, 24]. In the present study, univariable analysis revealed a linear correlation between L3MI and albumin and L3MI and CRP. However, the relationship between L3MI and albumin was not confirmed by multivariable analysis, which showed CRP was the only significant independent inflammatory markers correlated with L3MI. Although serum CRP is an agreed biomarker, cancer cachexia can be present in the absence of overt systemic inflammation [17]. In our cohort of SCLC patients, 85.2% of sarcopenic patients and 87.9% of non-sarcopenic patients had a normal CRP value. We suggest additional studies be conducted to determine the CRP value to be used as a surrogate marker of sarcopenia.

In patients with operable colorectal cancer, a direct relationship was reported between low skeletal muscle mass as determined by CT and mGPS [7]. CT provides an objective and reproducible means of quantifying skeletal muscle mass, and cross-sectional muscle area at L3 is regarded a gold standard for quantifying total body skeletal muscle mass [25]. Although sarcopenia is being increasingly recognized to be important for cancer patients, it is clear that the cutoffs used to evaluate skeletal muscle depletion in oncology patients vary considerably in clinical practice, and as a result, the implications of skeletal muscle depletion vary [4, 26]. In the present study we chose to use definitive cutoff values to define levels of sarcopenia as recommended by the international consensus of cancer cachexia [17], rather than study specific sex-specific quartile.

The present study has several limitations that require consideration. First, the number of patients included was small, although the study populations are homogeneous in terms of histology. The result of sarcopenia was significantly associated with male gender should be interpreted cautiously since small number of female patients (n = 30, 16.1%) was included. Second, the information such as pro-inflammatory cytokines cannot be obtained since this study was conducted in retrospective manner. Further in-vitro and in-vivo study would be needed for the verification of the relationship between skeletal muscle mass and pro-inflammatory cytokines for small cell lung cancer patients.

In conclusion, the present study confirms linear relationships between CT-determined skeletal muscle mass and serum CRP in patients with SCLC. These associations provide better understanding of the mechanism responsible for cancer cachexia, and hopefully, will aid the identification of novel drug targets and the development of better biomarkers for monitoring therapeutic efficacy.
